# CD39 delineates chimeric antigen receptor regulatory T cell subsets with distinct cytotoxic & regulatory functions against human islets

**DOI:** 10.3389/fimmu.2024.1415102

**Published:** 2024-06-28

**Authors:** Xiangni Wu, Pin-I Chen, Robert L. Whitener, Matthew S. MacDougall, Vy M. N. Coykendall, Hao Yan, Yong Bin Kim, William Harper, Shiva Pathak, Bettina P. Iliopoulou, Allison Hestor, Diane C. Saunders, Erick Spears, Jean Sévigny, David M. Maahs, Marina Basina, Seth A. Sharp, Anna L. Gloyn, Alvin C. Powers, Seung K. Kim, Kent P. Jensen, Everett H. Meyer

**Affiliations:** ^1^ Department of Medicine, Division of Blood and Bone Marrow Transplantation and Cell Therapy, Stanford University School of Medicine, Stanford, CA, United States; ^2^ Department of Internal Medicine, University of Missouri Kansas City, Kansas City, MO, United States; ^3^ Department of Developmental Biology, Stanford University School of Medicine, Stanford, CA, United States; ^4^ Departments of Medicine and of Pediatrics, Stanford University School of Medicine, Stanford, CA, United States; ^5^ Department of Chemical Engineering, Stanford University, Stanford, CA, United States; ^6^ Stanford Diabetes Research Center (SDRC), Stanford University School of Medicine, Stanford, CA, United States; ^7^ Division of Diabetes, Endocrinology and Metabolism, Department of Medicine, Vanderbilt University Medical Center, Nashville, TN, United States; ^8^ Centre de recherche du centre hospitalier universitaire (CHU) de Québec – Université Laval, Québec City, QC, Canada; ^9^ Départment de Microbiologie-Infectiologie et d’Immunologie, Faculté de Médecine, Université Laval, Québec City, QC, Canada; ^10^ Department of Pediatrics, Division of Pediatric Endocrinology, Stanford University School of Medicine, Stanford, CA, United States; ^11^ Department of Medicine, Division of Endocrinology, Gerontology, and Metabolism, Stanford University School of Medicine, Stanford, CA, United States; ^12^ Department of Genetics, Stanford University School of Medicine, Stanford, CA, United States; ^13^ Veterans Affairs (VA) Tennessee Valley Healthcare System, Nashville, TN, United States; ^14^ The Juvenile Diabetes Research Foundation (JDRF) Northern California Center of Excellence, Stanford University School of Medicine, Stanford, CA, United States; ^15^ Stanford Department of Medicine, Stanford, CA, United States; ^16^ Department of Pediatrics, Division of Stem Cell Transplantation, Stanford University School of Medicine, Stanford, CA, United States; ^17^ Department of Surgery, Abdominal Transplantation, Stanford University School of Medicine, Stanford, CA, United States

**Keywords:** Treg-regulatory T cell, chimeric antigen receptor, type 1 diabetes, immunoregulation, cytotoxicity

## Abstract

Human regulatory T cells (Treg) suppress other immune cells. Their dysfunction contributes to the pathophysiology of autoimmune diseases, including type 1 diabetes (T1D). Infusion of Tregs is being clinically evaluated as a novel way to prevent or treat T1D. Genetic modification of Tregs, most notably through the introduction of a chimeric antigen receptor (CAR) targeting Tregs to pancreatic islets, may improve their efficacy. We evaluated CAR targeting of human Tregs to monocytes, a human β cell line and human islet β cells *in vitro*. Targeting of HLA-A2-CAR (A2-CAR) bulk Tregs to HLA-A2^+^ cells resulted in dichotomous cytotoxic killing of human monocytes and islet β cells. In exploring subsets and mechanisms that may explain this pattern, we found that CD39 expression segregated CAR Treg cytotoxicity. CAR Tregs from individuals with more CD39^low/-^ Tregs and from individuals with genetic polymorphism associated with lower CD39 expression (rs10748643) had more cytotoxicity. Isolated CD39^−^ CAR Tregs had elevated granzyme B expression and cytotoxicity compared to the CD39^+^ CAR Treg subset. Genetic overexpression of CD39 in CD39^low^ CAR Tregs reduced their cytotoxicity. Importantly, β cells upregulated protein surface expression of PD-L1 and PD-L2 in response to A2-CAR Tregs. Blockade of PD-L1/PD-L2 increased β cell death in A2-CAR Treg co-cultures suggesting that the PD-1/PD-L1 pathway is important in protecting islet β cells in the setting of CAR immunotherapy. In summary, introduction of CAR can enhance biological differences in subsets of Tregs. CD39^+^ Tregs represent a safer choice for CAR Treg therapies targeting tissues for tolerance induction.

## Introduction

Regulatory T cells, described as CD3^+^CD4^+^CD25^+^CD127^low^FOXP3^+^ cells (Treg), are a rare population of immune cells that help control or prevent autoimmune diseases, including type 1 diabetes (T1D) (1, 2). Alterations in Treg function are associated with T1D progression. Preclinical studies suggest that increasing Treg numbers or function may provide a new way of treating T1D (3, 4). Multiple clinical trials demonstrate the safety of administering autologous, exogenously-expanded Tregs to recently diagnosed T1D patients (5, 6). In addition, current trials are underway to determine if transplantation of donor islets with infusion of autologous *ex vivo* expanded polyclonal Tregs may induce tolerance to islet allografts and prevent allogeneic rejection, while reducing the need for long-term immunosuppression (3, 7).

Augmentation of Treg function through genetic modification has emerged as a strategy to reduce or eliminate systemic immunosuppression following islet transplantation in preclinical and clinical studies. One promising Treg modification involves expression of chimeric antigen receptors (CARs). CARs are engineered proteins comprised of an extracellular antibody-derived antigen-binding domain (scFv) linked to intracellular signaling domains derived from CD3ζ and CD28. CARs can enhance the strength of Treg signaling, in parallel to the TCR complex (8). Thus, recognition of cognate antigen by CAR molecules can induce T cell activation in the absence of endogenous TCR signaling.

Preclinical studies show that CAR Tregs facilitate immune tolerance in islet transplantation and other models (5, 9, 10). Through cell surface CARs, Tregs can be directed to specific proteins on antigen presenting cells (APCs) or pancreatic islet β cells. In murine islet transplantation, transient CAR expression can target Tregs to donor islets and induce transient alloantigen specific tolerance (9). It is possible that sustained targeting of islet allografts could provide more durable alloantigen specific tolerance, but little is known about the long-term phenotypes resulting from stable expression of CARs in Tregs engineered to target islets.

Current understanding of CAR Treg function largely derives from studies of expanded total/bulk Treg populations. However, ‘bulk’ Tregs are comprised of heterogenous subpopulations, including some with cytotoxic potential. While CAR Tregs have been shown to kill APCs directly in specific settings (11, 12), it is unknown if CAR Tregs could kill islet β cells or other cell types, and how CAR expression might increase the risk of cytotoxicity in Treg subsets.

CD39 is a membrane-bound protein marker of a Treg subset associated with increased suppressive capacity and phenotypic stability (13). CD39 converts extracellular ATP or ADP to AMP; subsequently, CD73 converts AMP to adenosine, which has anti-inflammatory properties (14). In genome-wide association studies (GWAS) of autoimmunity, prior work has linked a specific genetic variation at rs10748643 with CD39 expression on immune cells, including Tregs (15, 16). Moreover, compared to healthy controls, subjects with T1D have fewer CD39^+^ Tregs (17). We therefore hypothesized that CD39 expression may correlate with reduced CAR Treg cytotoxic potential.

To test this hypothesis, here we evaluated CAR targeting of human Tregs to human monocytes, a pancreatic β cell line βlox5 (18), and human islet β cells *in vitro*. CAR Tregs targeting HLA-A2^+^ cells resulted in killing of human monocytes and islet β cells, and this cytotoxicity correlated *inversely* with CD39 expression by Tregs. Lentiviral transduction of CD39 in CD39^low^ Tregs to increase its expression reduced cytotoxic killing of βlox5 cells. Conversely, ablation of CD39 via CRISPR/Cas9-mediated genetic deletion increased βlox5 killing. An evaluation of CAR Tregs derived from FACS-sorted CD39^+^ and CD39^−^ populations confirmed key differences in cytotoxicity against monocytes, βlox5 cells, and primary human islet β cells. Both CD39^+^ and CD39^−^ CAR Treg populations induce strong islet cell expression of PD-L1 and PD-L2, and we found CAR Treg-dependent killing is modulated by the PD-1 and PD-L1/2 pathway. We also tested a novel CAR targeting of the recently described islet β cell surface marker NTPDase3 (19) (NTP-CAR). Like A2-CAR Tregs, NTP-CAR Tregs could be activated *in vitro*, but had significantly reduced cytotoxicity towards primary human islet β cells, suggesting that CAR Treg cytotoxicity could be regulated by specific CAR/ligand interactions. Thus, our work identified CD39-dependent phenotypes in CAR Tregs, and revealed that CD39^+^ Tregs may represent a better choice for therapeutic islet targeting strategies in T1D.

## Results

### HLA-A2-CAR Tregs derived from a subset of human donors are cytotoxic to monocytes

**Figure 1:**
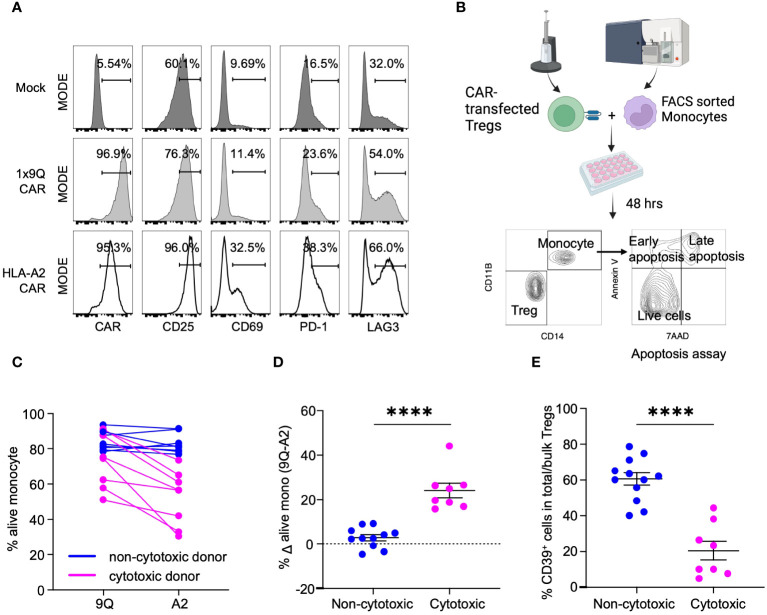
CAR Treg cytotoxicity against monocytes is dichotomous with Tregs from CD39^low^ donors being more cytotoxic. **(A)** Validation of HLA-A2-CAR expression and specificity toward HLA-A2 antigens. Tregs were mock transfected or transfected with HLA-A2 or control (1x9Q, 9Q) CARs and co-cultured with HLA-A2 (A2) positive monocytes. Activation of Tregs by A2-CARs were evaluated by surface markers, CD25, CD69, PD-1 and LAG3. **(B)** An experimental workflow to examine the cytotoxicity of A2-CAR Tregs towards A2 positive monocytes. **(C)** Viability of monocytes targeted by A2-CAR Tregs from different donors showed a bimodal distribution. The graph represents the percent of live (7-AAD^−^ Annexin V^−^) monocytes co-cultured with 9Q- or A2-CAR Tregs (n=12). A2-CAR Tregs from donors reduced monocyte viability were defined as cytotoxic (magenta), while the other donors were defined as non-cytotoxic (blue). **(D)** The viability difference between monocytes co-cultured with 9Q- and A2-CAR Tregs is calculated to determine the cytotoxicity. Percent Δ alive mono (Δ viability) is the % live monocyte co-cultured with 9Q-CAR Tregs subtracted by the % live monocyte co-cultured with A2-CAR Tregs. Blue indicates Δ viability <10%; magenta indicates increased cytotoxicity with a Δ viability ≥ 10%. **(E)** Cytotoxic Treg cohort showed lower percentage of CD39^+^ cells. Percentage of CD39^+^ cells within bulk Tregs was compared between cytotoxic (magenta) and non-cytotoxic donor cohorts (blue). **(D, E)** Data are presented as mean ± SEM, from n= 8-10 per group. ****P < 0.0001; by Student’s *t* test.

Human leukocyte antigen (HLA) class I molecules are constitutively expressed on nearly all human cells, and HLA-A2 is a common subtype in the US population (20, 21). Hence, HLA-A2 has been widely used as a target to study CAR Tregs (11, 22, 23). Here, we used an HLA-A2-CAR (A2-CAR) comprised of an anti-HLA-A2 scFv linked to CD28 and CD3ζ human stimulatory domains (**Supplementary Figure S1**) to determine if CAR Tregs could kill human monocytes. We transfected CARs into Tregs from healthy donors or patients with T1D and co-cultured them with allogeneic human monocytes. A control CAR, 1x9Q-CAR (9Q-CAR), targeting molecular conjugate FITC, was used as a negative control (9). Following electroporation, we confirmed that >90% of Tregs expressed the A2-CAR using HLA-A2-tetramers in flow cytometry (**Figure 1A**: Methods). A2-CAR Treg specificity was tested by co-culturing with HLA-A2^+^ monocytes. Activation of the Tregs induces CD25, CD69 and CD223 (LAG3), and flow cytometric analysis confirmed increased levels of these activation markers in A2-CAR Tregs compared to mock transfected and 9Q-CAR Treg controls (**Figure 1A**).

We then co-cultured A2-CAR Tregs derived from both healthy and T1D donors with HLA-A2^+^ human monocytes for 48 hours (**Figure 1B**). A dichotomous cytotoxic killing of monocytes was observed (**Figure 1C**). Co-culture of human monocytes with A2-CAR Tregs from a subset of donors led to a substantial reduction in monocyte viability. The differences of viable monocytes after co-culturing with A2- and 9Q-CAR Tregs was evaluated. We defined A2-CAR Tregs causing >10% reduction of viable monocytes as ‘cytotoxic’, whereas the others are ‘non-cytotoxic’ (**Figure 1D**). Our finding of donor-dependent A2-CAR Treg cytotoxicity provided an opportunity to investigate this phenotype.

We postulated that a Treg subpopulation might account for donor-dependent differences in CAR Treg cytotoxicity. We studied the cellular receptors common to Tregs by flow cytometry to identify markers distinguishing cytotoxic from non-cytotoxic subsets of Tregs and found that bulk Tregs with low frequency of CD39^+^ cells (CD39^low^ Tregs: **Supplementary Figure S2A**) were significantly more cytotoxic towards monocytes, compared to bulk Tregs with higher CD39 expression/frequency (CD39^high^ Tregs) (**Figure 1E**). This suggested that CD39 expression may regulate CAR Treg cytotoxicity.

### FACS-sorted and expanded CD39^+^ CAR Treg population is less cytotoxic

**Figure 2:**
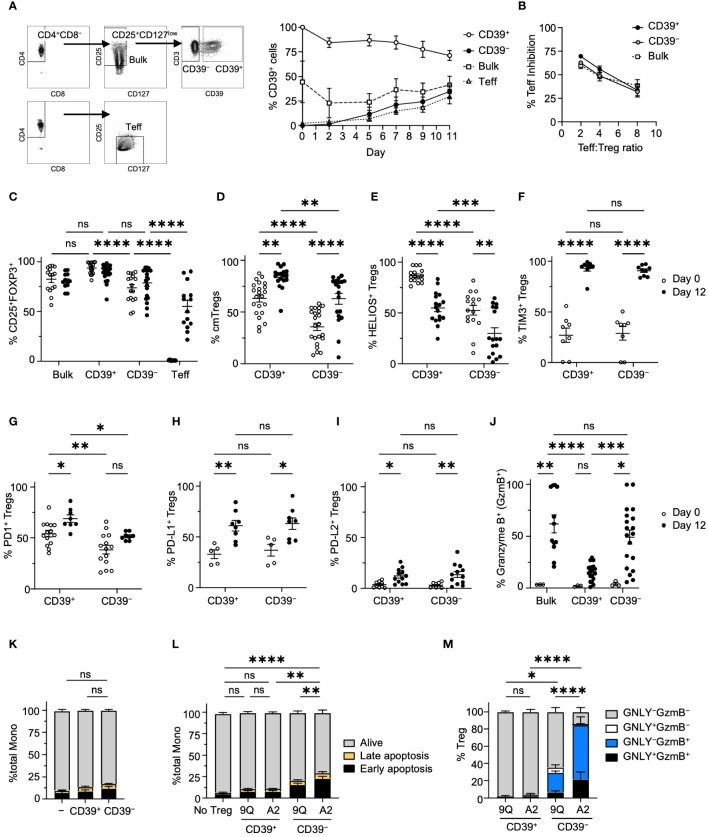
Characterization of CD39^+^ and CD39^−^ Treg subsets showed that CD39^+^ Tregs expressed higher levels of FOXP3 and lower Granzyme B. **(A)** The gating hierarchy to sort Teff, bulk Tregs and CD39^+^ and CD39^−^ Treg subsets. Adjacent graph showed the proportion of CD39^+^ cells in each subset during expansion. (mean ± SEM, n = 4). **(B)** MLR suppression assay among bulk, CD39^+^ and CD39^−^ Tregs. Each expanded Treg subset and CD4^+^ Teff cells were labeled and co-cultured as described in Materials and Methods, and the proliferation of Teff was analyzed by flow cytometry with the percent of Teff inhibition shown. (mean ± SEM, n = 3–4). **(C)** Percentage of CD25^+^FOXP3^+^ cells among bulk, CD39^+^, CD39^−^ Tregs and Teff cells on day 0 and 12 of cell expansion (mean ± SEM, n =12–20). **(D–I)** Percentage of CD45RO^+^ CD62L^+^ central memory(cm), HELIOS^+^, Tim3^+^, PD-1^+^, PD-L1^+^ or PD-L2^+^ cells in CD39^+^ and CD39^−^ Tregs on day 0 and 12 of cell expansion. (mean ± SEM, n =5–20). **(J)** Percentage of Granzyme B^+^ cells among CD39^+^, CD39^−^ and bulk Tregs on day 0 and 12 of cell expansion. (mean ± SEM, n =3–19). **(K)** Culture expanded CD39^+^ and CD39^−^ Tregs do not show increased cytotoxicity against allogeneic monocytes after co-culture. **(L)** A2-CAR transduced CD39^−^ Tregs showed increased cytotoxicity against allogeneic HLA-A2^+^ monocytes after co-culture. Cells were analyzed for early apoptotic (AnnexinV^+^7-AAD^−^), late apoptotic (AnnexinV^+^7-AAD^+^), and alive (AnnexinV^−^7-AAD^−^) cell populations. **(M)** Percentage of Granzyme B^+^ (GzmB^+^) and/or granulysin^+^ (GNLY^+^) cells in co-cultured CAR Tregs in L. Stacked bar plots represent mean ± SEM, n = 3–7. *P < 0.05, **P < 0.01, ***P < 0.001, ****P < 0.0001; ns, not significant by two-way ANOVA, Tukey’s multiple comparisons test.

To evaluate the association of CD39 expression with cytotoxicity in CAR Tregs, we isolated primary Tregs from individual donors to >95% purity by FACS as ‘bulk’, ‘CD39^+^’, and ‘CD39^−^’ Treg fractions and expanded these *in vitro* (**Figure 2A**; **Supplementary Figure S2B**). During cell expansion, bulk Tregs appeared to retain the same level of CD39 expression, whereas sorted CD39^+^ and CD39^−^ Tregs showed a mild decrease and increase of CD39 expression, respectively (**Figure 2A**). Expanded but unmodified bulk, CD39^+^, and CD39^−^ Tregs exhibited similar suppressive function (**Figure 2B**). As previously reported (13), primary, freshly-isolated bulk and CD39^−^ Tregs had lower FOXP3 expression compared to CD39^+^ Tregs (**Figure 2C**). Marker analysis revealed that primary CD39^+^ Tregs were predominantly central memory (CD62L^+^CD45RO^+^) Tregs, whereas <50% of primary CD39^−^ Tregs showed a central memory phenotype, suggesting that CD39^−^ Tregs are less differentiated compared to CD39^+^ Tregs (**Figure 2D**). Following culture expansion, both Treg subsets increased in central memory phenotype. While expression of Helios, a marker of Treg stability (24), was significantly higher in CD39^+^ cells, both subsets decreased expression over time (**Figure 2E**). Conversely, both CD39^+^ and CD39^−^ Treg subsets increased their expression of Tim-3, PD-1, PD-L1 and PD-L2 with culture expansion (**Figures 2F–I**). Interactions between Tim-3 and its ligand phosphatidylserine (PS), found on the surface of dying cells, lead to activation of Tregs and increased Treg suppressive function (25, 26). The increased expression of Tim3, PD-1, PD-L1 and PD-L2 suggests that these pathways may play an important role in expanded Treg suppressive function.

We then tested if cytotoxic effector molecules were different among CD39^+^ and CD39^−^ Treg subsets. Only 5% of primary bulk, CD39^+^, and CD39^−^ Tregs expressed granzyme B (GzmB), a regulator of perforin-dependent cytotoxicity against allogenic target cells (27). After expansion, however, >50% of bulk and CD39^−^ Tregs expressed GzmB. In contrast, GzmB expression remained low in CD39^+^ Tregs (**Figure 2J**). Despite noted differences in GzmB expression, when unmodified CD39^+^ or CD39^−^ Tregs were co-cultured with allogeneic monocytes, no significant increase in monocyte apoptosis was observed (**Figure 2K**). However, introducing A2-CAR into CD39^−^ Tregs significantly increased their activity. This was not observed with CD39^+^ CAR Tregs (**Figure 2L**). Of note, expression of A2-CAR in CD39^−^ Tregs resulted in higher levels of cytotoxic activity and cytotoxic effector molecule expression when compared to CD39^−^ Tregs expressing the irrelevant 9Q-CAR in co-cultures with HLA-A2+ monocytes (**Figure 2M**). Thus, our analysis revealed that CD39 expression were linked to distinct phenotypes contributing to the cytotoxicity in CAR Tregs.

### CD39^−^ CAR Tregs are more cytotoxic against the human β cell line, βlox5

**Figure 3:**
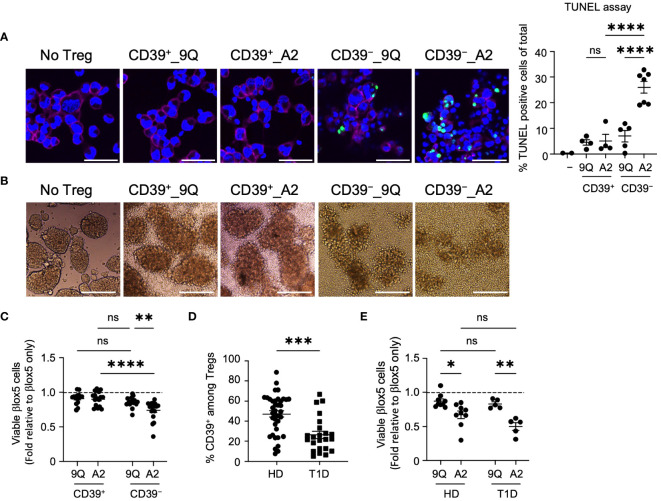
HLA-A2-CAR CD39^−^ Tregs can exert cytotoxic killing of human pancreatic β cell-line, βlox5. **(A)** Representative confocal microscopic images of cytotoxic killing. HLA-A2^+^ βlox5 cells were plated on coverslips overnight, and then 9Q- or A2-CAR transfected Tregs were added to co-culture for 24 hours. Cells were immuno-stained for HLA-A2 (red, βlox5 cells) and DAPI (blue), and then examined for apoptosis using TUNEL (green). % TUNEL positive cells of total cells per image are shown. Scale bar: 50 μm. (mean ± SEM, n= 2–7) **(B)** Representative microscopic images showing differences in βlox5 cell clustering with CAR Treg co-culture. 9Q- or A2-CAR transfected Tregs were added to βlox5 plated on a non-tissue culture treated 24-well plate for 48 hours and then imaged. Scale Bar: 200 μm. **(C)** CD39^+^ and CD39^−^ Tregs transfected with 9Q- or A2-CARs exert cytotoxic killing of βlox5 cells. Co-cultures of βlox5 cells and 9Q- or A2-CAR Tregs were set up as described in Materials and Methods. Viability was assessed by 7-AAD/Annexin V staining and flow cytometry. Percent of viable cells (AnnexinV^−^7-AAD^−^) were normalized and presented as fold relative to untreated βlox5 cells. (mean ± SEM, n= 15–18) **(D)** CD39 percentage gated on CD3^+^CD4^+^CD25^high^CD127^low^ Treg cells by flow cytometry in PBMCs from healthy donors (HD) and T1D patients. (mean ± SEM, n= 38 HD and 23 T1D) **(E)** Expanded CAR Tregs derived from both healthy donors (HD) and T1D patients show enhanced cytotoxic killing of βlox5 cells. βlox5 and HD or T1D CAR Treg co-cultures were set up and analyzed as in **(C)** (mean ± SEM, n= 9 HD and 5 T1D). *P < 0.05, **P < 0.01, ***P < 0.001, ****P < 0.0001; ns, not significant by one-way ANOVA in **(A)**, two-way ANOVA in **(C, E)** Tukey’s multiple comparisons test and by Mann-Whitney test in **(D)**.

To determine if our findings with CAR Tregs and monocyte killing extend to other cell types important in T1D, we co-cultured CAR Treg subsets with a human β cell line and primary cells, then assessed viability. We first evaluated Treg interactions with an immortalized cell line derived from human primary β cells, βlox5, which expresses HLA-A2. There was no significant change in viability of βlox5 cells co-cultured with unmodified CD39^+^ or CD39^−^ Tregs (**Supplementary Figure S3A**), whereas co-culture with A2-CAR Tregs increased βlox5 cell apoptosis (**Supplementary Figure S3B**). We visualized apoptosis of βlox5 cells with TUNEL (deoxynucleotidyl transferase dUTP nick end labeling) assay after removing CAR Tregs (**Figure 3A**). TUNEL staining was significantly higher in βlox5 cells co-cultured with CD39^−^ A2-CAR Tregs compared to the CD39^+^ A2-CAR and 9Q-CAR Treg control groups (**Figure 3A**). Like native islet cells, dispersed βlox5 spontaneously aggregate to form pseudoislets (**Figure 3B**) (28). When we evaluated βlox5 cell and CAR Treg co-cultures by light microscopy, we found that CD39^−^ A2-CAR Tregs disrupted the formation of pseudoislet-like clusters (**Figure 3B**), whereas CD39^+^ A2-CAR and 9Q-CAR Tregs did not.

We also quantified cytotoxicity of A2-CAR Treg subsets using flow cytometric analysis of 7AAD/Annexin V staining. This revealed that βlox5 cell viability was significantly reduced when co-cultured with CD39^−^ A2-CAR Tregs compared to the 9Q-CAR groups or CD39^+^ A2-CAR Tregs (**Figure 3C**). Consistent with previous reports (17), we analyzed peripheral blood mononuclear cells (PBMCs) from T1D and healthy donors and found that individuals with T1D had fewer CD39^+^ Tregs compared to healthy donors (**Figure 3D**). To determine whether Tregs from T1D patients carry the same cytotoxic capacity, βlox5 cells were co-cultured with bulk A2-CAR Tregs from healthy donors or patients with T1D. We observed increased cytotoxicity of A2-CAR Tregs derived from both healthy and T1D donors towards βlox5 cells (**Figure 3E**). These data suggest that caution should be taken when expanding and introducing CARs into autologous bulk or CD39^−^ Tregs, like in T1D subjects undergoing islet transplantation.

### βlox5 cells upregulate PD-L1 and PD-L2 levels in response to HLA-A2-CAR Tregs

**Figure 4:**
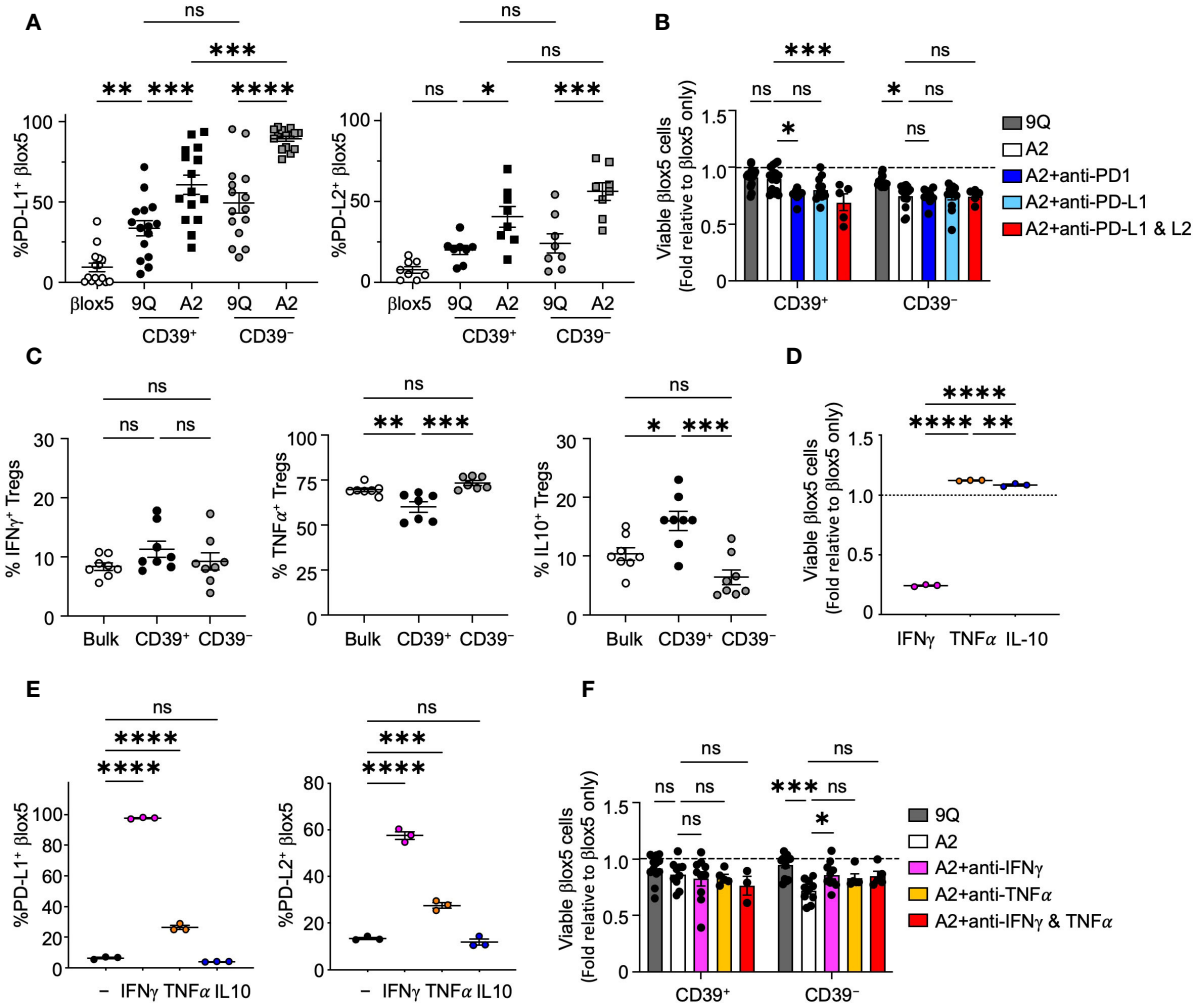
CAR Tregs induce PD-L1 and PD-L2 expression in βlox5 cells, which prevents CD39^+^ CAR Treg killing. **(A)** PD-L1 and PD-L2 expression in βlox5 cells co-cultured with CAR Tregs. 9Q- or A2-CAR transfected CD39^+^ and CD39^−^ Tregs were co-cultured with βlox5 cells for 24 hours. PD-L1 and PD-L2 expression were analyzed by flow cytometry in βlox5 cells (mean ± SEM, n=8–13). **(B)** PD-1/PD-L1 blockade increased cytotoxic killing of CD39^+^ CAR Tregs. CAR Treg and βlox5 cell co-cultures were set up as in **(A)** with or without PD-1 (50 µg/mL), PD–L1 (75 µg/mL) and/or PD–L2 (10 µg/mL) blocking antibodies. Viability was assessed by 7–AAD/Annexin V staining and flow cytometry. (mean ± SEM, n=5–14). **(C)** Cytokine production of bulk, CD39^+^ and CD39^−^ Tregs. Bulk, CD39^+^ and CD39^−^ Tregs were treated with PMA–Ionomycin for 4 hours, then proceeded with intracellular staining for IFN-γ, TNF-α and IL–10 (mean ± SEM, n=7–8). **(D)** βlox5 cells were treated with IFN-γ (20 ng/mL), TNF-α (20 ng/mL) or IL–10 (20 ng/mL) for 24 hours, and viability was assessed as in **(B)** (mean ± SEM, n=3). **(E)** βlox5 treated as in **(D)** were assessed for PD–L1 and PD–L2 expression (mean ± SEM, n=3). **(F)** CAR Treg and βlox5 cell co–cultures were set up as in **(A)** with or without blocking antibodies of IFN-γ (10 µg/mL) and/or TNFα (10 µg/mL), and viability was assessed. (mean ± SEM, n=3–14). *P < 0.05, **P < 0.01, ***P < 0.001, ****P < 0.0001, ns, not significant by one–way ANOVA, Tukey’s multiple comparisons test.

The cell surface proteins PD-L1 and PD-L2 can be expressed on diverse cell types to modify immune reactivity, including attenuation of T cell autoimmune destruction of islet β cells in T1D (29). To determine if β cells could increase expression of PD-L1 or PD-L2 to prevent cytotoxicity in response to CAR Tregs, we co-cultured A2-CAR CD39^+^ and CD39^−^ Tregs with βlox5 cells. We observed a significant upregulation of both PD-L1 and PD-L2 in βlox5 cells (**Figures 4A, B**) when compared to βlox5 co-cultured with 9Q-CAR Tregs. We also observed a corresponding increase in PD-1 expression by CD39^+^ and CD39^−^ A2-CAR Tregs (**Supplementary Figure S4**). While co-culture induced the expression of PD-1 in both CD39^+^ and CD39^−^ CAR-Tregs, higher levels were exhibited by CD39^+^ CAR Tregs.

To investigate the possibility that PD-1/PD-L1 signaling could modulate cytotoxic effects of A2-CAR Tregs, we added blocking antibodies specific for PD-1, PD-L1 or PD-L2 separately or in combination to βlox5 and CAR Treg co-cultures. Blockade of PD-1 or PD-L1 and PD-L2 resulted in a significant decrease in viability of βlox5 cells co-cultured with A2-CAR CD39^+^ Tregs. By contrast, blocking these pathways had no effect on βlox5 viability when co-cultured with CD39^−^ A2-CAR Tregs (**Figure 4B**). This suggested that CD39^+^ CAR Tregs respond to signaling of the PD-1, PD-L1/PD-L2 pathways by reducing cytotoxic activity, but CD39^−^ CAR Tregs appear less sensitive to this immunoregulation.

### CD39^+^ and CD39^−^ Tregs produce cytokines that induce PD-L1 and PD-L2 expression

Tregs subsets can produce immunoregulatory cytokines, such as IL-10 (30), as well as cytokines like IFN-γ and TNF-α that can induce PD-L1 and PD-L2 expression (31, 32). Both IFN-γ and TNF-α are also cytotoxic to islet β cells (33, 34). We therefore evaluated the cytokine profile of CD39^+^ and CD39^−^ Tregs by intracellular cytokine staining after Phorbol Myristate Acetate (PMA) and ionomycin stimulation. We found that both CD39^+^ and CD39^−^ Tregs are capable of IL-10, IFN-γ and TNF-α production (**Figure 4C**). While there was no significant difference in the proportion of cells producing IFN-γ between CD39^+^ and CD39^−^ Tregs, significantly more CD39^−^ Tregs produced TNF-α compared to CD39^+^ Tregs. CD39^+^ Tregs produced significantly more IL-10 compared to CD39^−^ Tregs (**Figure 4C**). When we treated βlox5 cells with IFN-γ, TNF-α or IL-10, we found that IFN-γ but not TNF-α or IL-10 significantly reduced the viability of βlox5 cells *in vitro* (**Figure 4D**). Both IFN-γ and TNF-α induced expression of PD-L1 and PD-L2 in βlox5 cells (**Figure 4E**). Addition of IFN-γ blocking antibody to A2-CAR Treg and βlox5 cell co-cultures reduced the cytotoxicity of A2-CAR CD39^−^ Tregs, while blockade of TNF-α had no effect on cytotoxicity (**Figure 4F**). Together with other results (**Figure 3**), these data demonstrate that A2-CAR CD39^−^ Tregs may kill βlox5 cells via IFN-γ mediated pathways.

### Genetic variation associated with altered CD39 expression correlates with Treg cytotoxicity

**Figure 5:**
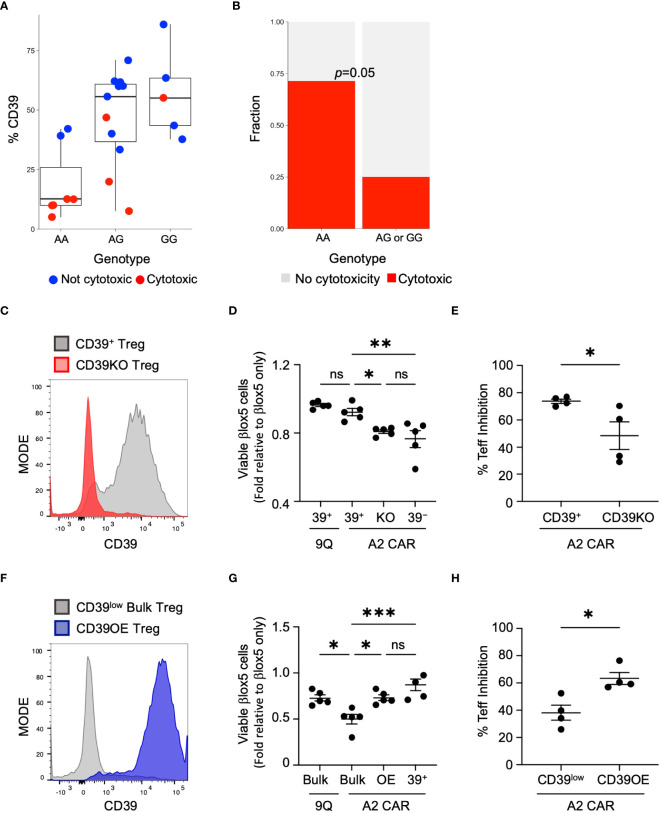
CD39 expression and cytotoxicity varies by donor genotype at rs10748643 and manipulation of CD39 levels alters cytotoxicity and suppression activity. **(A)** Genotype of healthy and T1D donors at rs10748643 obtained via SNP microarray was correlated with CD39 expression and CAR Treg cytotoxicity. **(B)** Cytotoxicity with CAR Tregs was specifically enriched in donors with the AA genotype at rs10748643. *P* value by Fisher Exact Test. **(C)** Knockout of CD39 increases cytotoxic potential. CD39^+^ Tregs was knocked out of CD39 by CRISPR–Cas9 and tested for knockout efficiency 4–5 days post–transfection. **(D)** CD39^+^, CD39KO (KO) and CD39^−^ Tregs were transfected with 9Q– or A2–CARs as indicated and co–cultured with βlox5 cells. Cell viability was assessed by flow cytometry. (mean ± SEM, n=5). **(E)** Mixed lymphocyte suppression assays with A2–CAR transfected CD39^+^ and CD39KO (KO) Tregs were set up as described in Materials and Methods at a Treg: Teff ratio of 1:2 and analyzed. (mean ± SEM, n=4). **(F)** Bulk Tregs from a CD39^low^ donor were lentiviral transduced to over–express CD39 and GFP. FACS–enriched GFP^+^ Tregs were expanded and analyzed for stable expression of CD39 (CD39OE). **(G)** Overexpression of CD39 reduces cytotoxic killing of CAR Tregs against βlox5. CD39^low^ bulk, CD39OE, and CD39^−^ Tregs were transfected with 9Q–, or A2–CARs as indicated, co–cultured and analyzed as in **(D)** (mean ± SEM, n=5). **(H)** Suppression activities of A2–CAR transfected CD39^low^ and CD39OE bulk Tregs were set up and analyzed as in **(E)** *P < 0.05, **P < 0.01, ***P < 0.001, ns, not significant by one–way ANOVA, Tukey’s multiple comparisons test in **(D, G)** and by Student’s *t* test in **(G, H)**.

Genetic variation at the *CD39* locus can influence CD39 transcript levels (15, 16). This has been demonstrated specifically in Tregs for variant rs10748643 located in *CD39* intron 1 (16). We reasoned that if *CD39* expression is genetically determined, then increased CAR Treg cytotoxicity might co-segregate with alleles linked to decreased CD39 levels. To assess this, we isolated and sequenced genomic DNA from 26 individuals including T1D and healthy controls to assess the presence of variant rs1074864. We found that individuals homozygous for guanine (G/G) at rs10748643 exhibited a high frequency of CD39^+^ Tregs. Individuals heterozygous (A/G) at rs10748643 have intermediate expression, and individuals homozygous for adenine (A/A) have reduced or low expression of CD39 protein (16) (**Figure 5A**). We found that the A/A genotype, that correlated with low frequency of CD39^+^ Tregs, was enriched for cytotoxicity (*P*=0.05, Fischer’s exact test: **Figure 5B**). Thus, genetic variation at the *CD39* locus influenced CD39 expression and CAR Treg cytotoxicity.

### Genetic modulation of CD39 expression regulates CAR Treg cytotoxicity

If expression of CD39 is a determining factor in Treg cytotoxicity, then loss of CD39 expression by A2-CAR CD39^+^ Tregs could result in increased cytotoxicity against islet cells. To test this hypothesis, we used CRISPR/Cas9 to knock out *CD39* expression in CD39^+^ Tregs (CD39KO) prior to the introduction of the A2-CAR (**Figure 5C**). When βlox5 cells were co-cultured with A2-CAR CD39KO Tregs, the viability of βlox5 cells was significantly reduced (**Figure 5D**). Additionally, mixed lymphocyte suppression assay showed that knockout of CD39 expression reduced the suppressive function of A2-CAR Tregs (**Figure 5E**).

To further investigate links between CD39 expression and CAR Treg cytotoxicity, we tested whether induction of CD39 expression could reverse the cytotoxicity of A2-CAR bulk Tregs expressing low levels of CD39 (CD39^low^ Tregs). Using CD39 lentiviral transduction, we over-expressed CD39 in CD39^low^ Tregs (CD39OE Tregs) (**Figure 5F**). When βlox5 cells were co-cultured with A2-CAR CD39OE Tregs, the viability of βlox5 cells was significantly increased (**Figure 5G**). Likewise, the suppressive function of A2-CAR CD39OE Tregs was improved (**Figure 5H**). These findings are consistent with our genomic SNP data (**Figure 5B**) and show how genetic modification to achieve *CD39* expression is sufficient to suppress cytotoxicity of CAR Tregs.

### HLA-A2-CAR CD39^−^ Tregs are cytotoxic to primary human islet β cells

**Figure 6:**
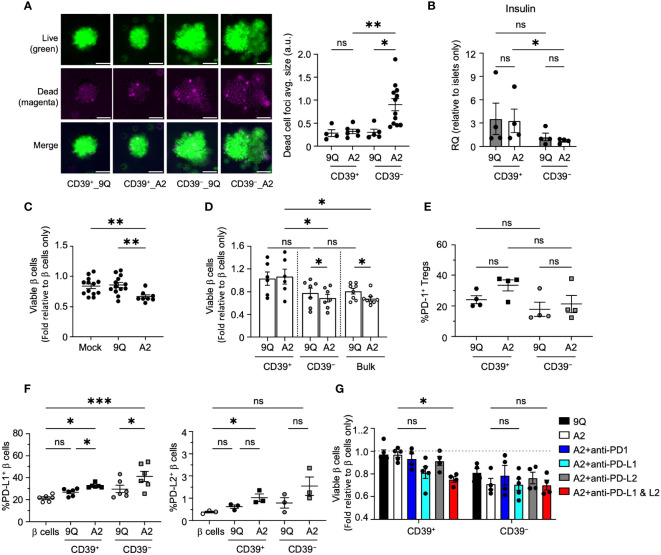
CD39^+^ A2–CAR Tregs are less cytotoxic to primary human islet β cells, which depends on the PD–L1 pathway to prevent cytotoxicity. **(A)** Microscopic evaluation of HLA–A2^+^ primary islets’ response to CAR Tregs. Representative images of intact human islets co–cultured with indicated CAR Tregs for 48 hours. Live/dead islet cells were stained with calcein–AM (green)/ethidium homodimer–1(magenta) and imaged by fluorescent microscope. Average size of dead cell foci per image per condition was analyzed using ImageJ and graphed in adjacent scatter plot. Scale bar: 50 μm. (mean ± SEM, n=4–12 images) **(B)** Insulin (*INS*) gene expression. Intact human islet–Treg co–cultures set up as in A for 24 hours. Post co–cultured islets were collected. RNA was extracted and assessed for insulin gene expression using quantitative RT–PCR. (mean ± SEM, n=4) **(C)** Flow cytometric evaluation of primary islet viability. HLA–A2^+^ human islets were dispersed into single cells and co–cultured with bulk CAR Tregs for two days and stained with HPi2, HPx1, 7–AAD and Annexin V. Percent of viable β cells (HPi2^+^ HPx1^−^ AnnexinV^−^7–AAD^−^) were normalized and presented as fold relative to untreated β cells. (mean ± SEM, n=8–13) **(D)** Evaluation of CD39^+^ and CD39^−^ CAR Treg cytotoxicity to primary islet β cells. Islet cells were co–cultured with 9Q– or A2–CAR transduced bulk, CD39^+^ and CD39^−^ Tregs for 48 hours and cell viability was assessed as in C (mean ± SEM, n=6–8). **(E, F)** Evaluation of the PD1/PD–L1/2 pathway. Islet cell co–cultures were set up with indicated CAR Tregs as above and analyzed for PD–1 expression in Tregs and PD–L1 and PD–L2 expression in β cells. (mean ± SEM, n=3–6). **(G)** CAR–Treg and islet cell co–cultures were set up as in D with or without PD–1 (10 µg/mL), PD–L1 (10 µg/mL) and/or PD–L2 (10 µg/mL) blocking antibodies and analyzed for islet cell viability (mean ± SEM, n=4–5). *P < 0.05, **P < 0.01, ***P < 0.001, ns, not significant by one–way ANOVA, Tukey’s multiple comparisons test in **(A, C, E–G)** and by Mann–Whitney test in **(B, D)**.

To confirm that these findings extend to primary human β cells, we obtained cadaveric HLA-A2^+^ primary human islets to perform co-cultures with 9Q-CAR or A2-CAR transfected Tregs (both CD39^+^ and CD39^−^ subsets). Live and dead cells in intact islets were stained by calcein-AM and ethidium homodimer-1, respectively, then imaged. A2-CAR CD39^−^, but not CD39^+^ Tregs, resulted in a significant increase of apoptotic cell death in primary human islets (**Figure 6A**). To assess primary islet function in co-cultures with A2-CAR Tregs, we measured islet mRNAs encoding insulin (**Figure 6B**). Compared to islets co-cultured with 9Q-CAR or A2-CAR CD39^+^ Tregs, islets co-cultured with A2-CAR CD39^−^ Tregs had a significant decrease in insulin (*INS*) expression (**Figure 6B**). We infer that the reduction of insulin expression was linked to β cell death owing to the cytolytic function of A2-CAR CD39^−^ Tregs.

To assess CAR Treg effects further, we co-cultured dissociated human islet cells with Tregs then quantified apoptotic endocrine cells via flow cytometry after 7AAD/Annexin V staining (**Supplementary Figure S5**). Consistent with our findings in intact islets, we found that addition of A2-CAR bulk or CD39^−^ Tregs to human islet cells significantly decreased endocrine cell viability (**Figures 6C, D**) but found no significant reduction of primary human islet cell viability when co-cultured with 9Q- or A2-CAR CD39^+^ Tregs (**Figure 6D**).

### PD-L1 and PD-L2 expression by primary human β cells is induced by HLA-A2-CAR Tregs

We next assessed PD-1/PD-L1 signaling in dissociated primary human islet cells co-cultured with CAR Tregs. We observed that both A2-CAR CD39^+^ and CD39^−^ Tregs induced expression of PD-L1 and PD-L2 in islet endocrine cells (**Figures 6E**); by contrast, PD-1 expression in A2-CAR CD39^+^ and CD39^−^ Tregs after co-culture (**Figure 6F**) was not significantly increased. Antibody blockade of PD-L1 and PD-L2 increased islet cell apoptosis targeted by A2-CAR CD39^+^ Tregs (**Figures 6G**). These data with primary human islet cells support findings using β cell lines and suggest a role for the PD-1/PD-L1/PD-L2 signaling in protection of human islet β cells by A2-CAR CD39^+^ Tregs.

### CAR Tregs targeting β cell NTPDase3 have reduced cytotoxicity compared to HLA-A2-CAR Tregs

Is CAR Treg cytotoxicity for β cells dependent on the CAR target? To assess this, we built a CAR to target Ectonucleoside Triphosphate Diphosphohydrolase-3 (NTPDase3), a cell surface enzyme highly enriched in adult human β cells (35). We constructed a novel CAR molecule targeting human NTPDase3 (NTP-CAR; Methods), based on the finding that a monoclonal antibody (Clone hN3-B3S) (36) targeting human NTPDase3 was found to successfully label human islets transplanted into anterior chamber of the eye of immunodeficient NSG mice when injected intravenously (35).

**Figure 7:**
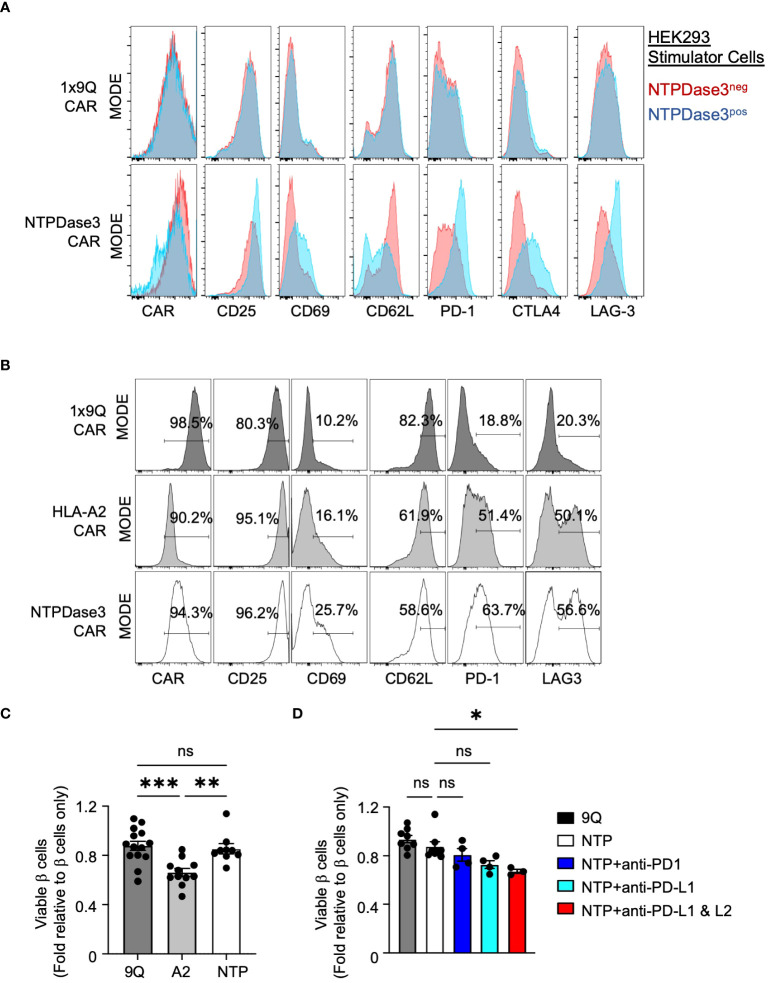
Alternative CAR Tregs targeting NTPDase3 (NTP–CAR) were less cytotoxic to human islet β cells. **(A)** Representative flow staining of 1x9Q–CAR (top row) or NTP–CAR Tregs (bottom row) co–cultured with HEK–293 cells either expressing NTPDase3 (Blue histograms) or not (Red histograms). Relative expression levels of CAR molecule, along with activation markers CD25 and CD69, as well as CD62L, PD–1, CTLA4, and LAG–3 are shown. **(B)** Representative flow staining of 1x9Q–CAR, A2–CAR, or NTP–CAR Tregs cultured with dispersed HLA–A2^+^ human islet cells. A2–CAR and NTP–CAR Tregs showed elevated levels of activation and suppressive markers compared to 9Q–CAR Tregs. **(C)** Flow cytometric evaluation of primary islet β cell viability after co–culturing with CAR Tregs. HLA–A2^+^ human islets were dispersed to single cells and co–cultured with bulk 9Q–CAR, A2–CAR, or NTP–CAR Tregs for 48 hours and stained for HPi2, HPx1, 7–AAD and Annexin V. Percent of viable β cells (HPi2^+^ HPx1^−^AnnexinV^−^7–AAD^−^) were normalized and presented as fold relative to untreated β cells. (mean ± SEM, n=9–14) **(D)** Evaluation of the PD1/PD–L1/2 pathway. NTP–CAR Treg and islet cell co–cultures were set up as in **(C)** with or without PD–1 (10 g/mL), PD–L1 (10 g/mL) and/or PD–L2 (10 g/mL) blocking antibodies and analyzed for β cell viability. (mean ± SEM, n=3–8) *P < 0.05, **P < 0.01, ***P < 0.001, ns, not significant by one–way ANOVA, Tukey’s multiple comparisons test in **(C, D)**.

To confirm the function of NTP-CAR, we used lentivirus to express NTPDase3 in HEK293T cells, which do not express detectable levels of endogenous NTPDase3 (Methods). NTPDase3^+^ HEK293T cells were enriched via FACS sorting and passaged. NTPDase3^−^ cells were also collected and passaged as negative control cells. These cells were then co-cultured in the presence of 9Q-CAR or NTPDase3-CAR Tregs, and the phenotypic activation status of the CAR Tregs was determined via flow cytometry. We observed no difference in phenotype of 9Q-CAR Tregs when cultured with either NTPDase3^−^ or NTPDase3^+^ cells. However, we observed marked reduction in surface NTP-CAR and CD62L levels on NTP-CAR Tregs when co-cultured with NTPDase3^+^ HEK293 cells as compared to NTPDase3^−^ HEK293 cells, suggesting a successful, and specific, CAR-ligand interaction. Additionally, only when NTPDase3-CAR Tregs were cultured with NTPDase3^+^ HEK293 cells, we also observed increased CAR Treg production of activation markers CD25 and CD69, as well as markers associated with suppressive function, such as PD-1, CTLA4, and LAG-3. Taken together, we conclude that the NTP-CAR is expressed, trafficked to the cell surface, and successfully initiates Treg activation when NTPDase3 is encountered on the surface of a target cell, even in the absence of endogenous TCR signaling (**Figure 7A**).

We then sought to determine whether NTP-CAR Tregs would recognize and respond to NTPDase3 expressed by primary human islet cells. We co-cultured primary HLA-A2^+^ human islet cells with either 9Q-CAR, A2-CAR, or NTP-CAR expressing Tregs. Both A2-CAR and NTP-CAR Treg populations demonstrated surface marker profiles indicative of activation, when compared to 9Q-CAR Tregs (**Figure 7B**). Interestingly, markers associated with activation (CD25, CD69) and suppressive function (PD-1, LAG-3) were more elevated in NTP-CAR Tregs when compared to their A2-CAR Tregs. This suggests that the NTP-CAR more efficiently activated the Tregs, although further studies are needed to address this.

To determine whether NTP-CAR Tregs would display similar cytotoxicity towards human islet cells as the A2-CAR Tregs, we co-cultured human islet cells with 9Q-CAR, A2-CAR, or NTP-CAR Tregs, and assessed islet cell viability with 7AAD/Annexin V staining in flow cytometry. Co-culture with A2-CAR Tregs resulted in reduced islet cell viability when compared to Tregs expressing 9Q-CAR. In striking contrast, we saw no difference in islet cell viability with NTP-CAR Tregs as compared to 9Q-CAR Tregs (**Figure 7C**), strongly suggesting that cytotoxic activity is dependent upon the strength of the activation signal transduced upon CAR/ligand interaction. Similar to findings with A2-CAR Tregs (**Figure 6**), this lack of cytotoxicity was dependent upon PD-L1/PD-L2 signaling: co-culturing NTP-CAR Tregs with primary human islets in the presence of PD-L1 and/or PD-L2 blocking antibodies resulted in reduced islet cell viability (**Figure 7D**). Together, these data suggested that NTP-CAR Tregs may form the basis of future islet-targeted CAR Treg strategies for T1D and islet transplantation.

## Discussion

Intensive efforts have focused on harnessing Treg cells for therapeutic strategies in recent-onset or established T1D, including islet transplantation (3, 7, 37). While genetic modification of Tregs through the introduction of chimeric antigen receptors can bolster their immuno-suppressive function - one desirable outcome for T1D and other autoimmune diseases (9, 38, 39) - little is known about human CAR Treg phenotypic stability or cytotoxic potential against islets. Work here reveals crucial differences in the cytotoxic potential of human CD39^+^ and CD39^−^ CAR Tregs against human islets, with important implications for islet-directed therapies.

CD39^+^ Tregs are a biologically distinct subset with features of central memory-like T cells, that retain higher levels of canonical natural Treg master transcription factors, HELIOS and FOXP3, and expand more slowly than their CD39^−^ counterparts. Both CD39^+^ and CD39^−^ populations can lose or re-express some CD39 during *ex vivo* expansion. While functional differences between CD39^+^ and CD39^−^ Treg subsets have been reported (13), our work reveals that these differences are enhanced by CAR transgenesis and targeting. For example, there are no significant differences in cytotoxic effects when co-culturing unmodified CD39^−^ versus CD39^+^ Treg subsets with allogeneic islets, but CD39^−^ CAR Tregs clearly have higher cytotoxic potential, likely due to increased expression of cytolytic molecules like granzyme B and granulysin. Furthermore, our data pinpoints CD39^−^ Tregs as being the principal contributors of cytotoxicity killing in bulk CAR Treg cell products.

We and others (17) find that subjects with T1D have fewer CD39^+^ Tregs compared to non-T1D controls. This has important implications for autologous Treg cell products derived from T1D patients: for example, our study - though not comprehensive - suggests that CAR Tregs from T1D patients are more cytotoxic than those from healthy donors. It is still unclear whether lower CD39 expression in T1D is due to genetic predisposition, or perhaps reflects inflammatory conditions in new onset T1D that could lower CD39 expression, which has been found in other disease settings (40). Further studies are needed to understand T1D Treg biology.

Based on its enzymatic functions in regulating ATP metabolism to yield immunoregulatory factors like adenosine, CD39 activity itself is immunoregulatory (14). In addition, our genetic loss- and gain-of-function studies address the possibility that CD39 is a key signaling regulator of cytotoxicity and immune suppression. Following CRISPR/Cas9 based knock-out of *CD39*, we found increased CAR Treg cytotoxicity and diminished suppressive capacity. In complementary findings with CD39 overexpression, we found that there was reduced cytotoxicity, and maintained immunosuppression. Together these data suggest that CD39 activity levels in CAR Tregs can regulate signaling to modulate Treg function and provide evidence that strategies to enhance *CD39* expression may be beneficial for targeted immunosuppression with CAR Tregs.

Genetic variation within the promoter region of *CD39* is known to alter CD39 expression in humans (16, 41) and here we report that the A/A genotype at rs10748643 within intron 1 was associated with reduced *CD39* expression in CAR Tregs and increased islet cytotoxicity. Thus, our work identifies a mechanism to stratify potential patient subsets that may benefit from autologous CAR Treg treatment. However, we also observed the variation in some CD39^hi^ Tregs with enhanced cytotoxicity, particularly in the Tregs derived from T1D patient samples. More comprehensive genetic screening of T1D patient samples is underway to uncover the co-factors that account for this variability and improve the clinical application of this finding. While targeted immunosuppression with CD39^+^ CAR Tregs could be used for auto-immune diseases like T1D, we speculate that our findings could also influence use of CD39^–^ cytotoxic CAR Tregs in other settings, like neuroendocrine cancer targeting.

There remains much to be explored and understood about the biology of CAR Tregs in the setting of T1D and other diseases. Our studies highlight the complex interplay between pro-inflammatory and immunoregulatory pathways, for example both CD39^+^ and CD39^−^ subsets produce IFN-γ and TNF-α which can be toxic to islets, and importantly the CD39^+^ subset may produce more IL-10. The production of these cytokines can directly drive the upregulated surface expression of PD-L1 and PD-L2 in βlox5 and primary islets. Blocking PD1-PD-L1/2 did not detectably alter apoptotic death in islets targeted by CD39^−^ CAR Tregs, suggesting that these cells are already maximally capable of cytotoxicity and not sensitive to immunoregulation by PD-L1 and PD-L2. Unexpectedly, CD39^+^ CAR Tregs stimulated apoptotic killing when PD-L1 and PD-L2 expression is blocked, suggesting that CD39^+^ Tregs require this pathway for optimal immunoregulation. It is important that this axis be evaluated in future studies especially when CAR Tregs may be integrated into combination immunotherapy that could affect IFN-γ, TNF-α or PD-L1/2 expression. Other cytokine production and differentially expressed molecules between CD39^+^ and CD39^−^ Tregs subsets, especially after co-culturing, are currently under investigation to further delineate the interaction of CD39 in Tregs and its cytotoxicity to target cells. Likewise, the differences between HLA-A2 and NTPDase3 targeting by CAR Tregs, especially differential cytotoxic potential against human islet cells, requires further study.

Our study has limitations, including the as-yet unstudied possibility that HLA-A2 and NTPDase3-based CAR targeting could be influenced by islet target protein density and dynamic regulation, or alter potential immunomodulatory functions of targets like NTPDase3 (19). Further studies reconstituting *in vivo* cell targeting could complement *in vitro* assays used here. Additional work could also delineate the signal transduction pathways linking CAR activation in CD39^+^ versus CD39^–^ Tregs, leading to immunosuppression or cytotoxic effects. The caveat that our study includes use of an immortalized human β cell line is offset by supportive findings in key studies using primary human islet cells.

In conclusion, our work identifies fundamental differences in the phenotype and function of CAR Treg subsets, with important implications for clinical transplantation. Furthermore, polymorphism in pathways like those that control *CD39* expression can shape the cytotoxic and immunoregulatory potential of bulk Treg cell cultures and CAR Tregs. CD39^+^ Tregs may be a preferable Treg subset for use in CAR Treg cell therapies to protect human islets or other tissue targets in future clinical applications.

## Materials and methods

### Patients and disease status

T1D patients provided informed consent in accordance with the Declaration of Helsinki and were enrolled in a research protocol approved by the Stanford University Institutional Review Board (IRB) protocol #35453. Patients were clinically evaluated for T1D, and auto-antibody reactivity was verified by clinical testing. For healthy controls, leukoreduction chambers or buffy coats were purchased from Stanford Blood Center (Palo Alto, CA). In this study, all Treg donors are derived from HLA-A2^neg^ donors. We did not match control donors with T1D patient samples.

### Primary human islets

Primary cadaveric human islets were obtained from the IIDP, ADI Islet Core, or the UCSF Diabetes Center Islet Production Core. Only islet preparations which were >75% pure based on dithizone (DTZ) staining and would arrive within 96 hours of isolation were selected.

### Cell purification, *in vitro* expansion, and characterization

Fresh peripheral blood mononuclear cells (PBMC) were isolated from healthy donors or from freshly drawn blood of patients with T1D by gradient centrifugation on Ficoll-Hypaque gradients (GE Healthcare Bioscience). CD3^+^CD4^+^CD25^high^CD127^low^ Tregs were isolated using the EasySep Human Regulatory T Cell Isolation Kit (StemCell Technologies) and sorted to >95% purity on a FACSAria III Cell Sorter (BD Biosciences). The gating strategy is provided in **Supplementary Figure S2B**. Conventional CD4 T cells were also sorted by FACS. The fluorophore-conjugated monoclonal antibodies used are listed in **Supplementary Table S1**. Tregs were cultured for 12 consecutive days, checked every 6 days for purity by flow cytometry. Details of cell culture expansion are described in **Supplementary Materials and Methods**. Treg purity was assessed by staining with anti-FOXP3 (Clone 206D, BioLegend) in eBioscience FOXP3 Transcription Factor staining buffer set (ThermoFisher). Flow cytometry data were collected on an LSRII (Becton Dickinson). FlowJo version v10 software (Treestar) was used for analysis.

### CAR constructs and production of CAR mRNA

HLA-A2-CAR construct was a kind gift from Dr James Riley, University of Pennsylvania (11). The NTPDase3-CAR construct was made by sequencing the variable segment of a monoclonal antibody specific for human NTPDase3 (clone hN3-B3s, Dr. Jean Sévigny), converting the sequence into the scFv form, and then cloning that sequence into the pCDH-EF1a-MCS vector. A CAR which recognizes the irrelevant antigen fluorescein isothiocyanate (1X9Q-CAR) was used as a control CAR (9). Linear mRNA molecules of each CAR for Treg transfection were produced using the T7 mScript™ Standard mRNA Production System (CellScript LLC, Madison, WI, USA).

### Transfection of Tregs

Prior to electroporation, expanded Tregs were activated again at day 9 with Dynabeads^®^ Human T-Activator CD3/CD28 in a bead: cell ratio = 1: 2 for 2–3 days in the presence of 500 IU/ml rhIL-2. On the day of transfection, activated Tregs were counted, bead-removed, washed with PBS, and resuspended in electroporation buffer. A total of 2–3×10^6^ Tregs were electroporated with 10–20 μg of CAR mRNAs using a Neon Transfection System (Invitrogen) under the following conditions: voltage (2400 V), width (10 ms), pulses (three), 100-μl tip, and Buffer T. Transfected cells were plated in 2–3 ml of antibiotics-free RPMI 1640 complete media (+500 IU rhIL-2/ml). The CAR transfection efficiency was checked using an anti-Flag mAb (anti-DYKDDDDK) or HLA-A2 Tetramer (NIH Tetramer Core Facility) after 24 hours.

### CAR-Treg and monocyte co-culture for flow cytometric analysis of monocyte apoptosis

Monocytes were enriched from PBMCs using EasySep Human Monocyte Isolation Kit (StemCell Technologies) and then sorted to purity gating on CD3^−^CD56^−^CD19^−^CD11b^+^CD14^+^HLA-DR^+^ population. Monocytes were co-cultured with CAR-Tregs in 48-well plates at a ratio of 1:1 and density of 1x10^6^ cells/ml each for 48 hours. Cells were collected and stained with FITC-Annexin V Apoptosis Detection Kit with 7-AAD (BioLegend) according to the manufacturer’s instructions. Samples were then analyzed by flow cytometry.

### CAR-Treg and βlox5 cell co-culture for flow cytometric analysis of βlox5 apoptosis and pseudo-islet imaging

βlox5 cells were a kind gift from Clayton Matthews (University of Florida). βlox5 culture conditions are provided in **Supplementary Materials and Methods**. 0.5x10^5^ βlox5 cells and 2x10^5^ CAR-Tregs were co-cultured in 96-well flat-bottom culture plate in a mixture of Treg and βlox5 media (1:1) in the presence of rIL-2 (100 IU/ml) for 24 hours. Co-cultured βox5 cells were harvested with TrypLE (ThermoFisher) for 3 minutes to resuspend single cells, and then stained with FITC-Annexin V Apoptosis Detection Kit with 7-AAD (BioLegend). Samples were then analyzed with flow cytometry.

For βlox5 pseudo-islet formation assay, 1x10^5^ βlox5 cells and 4x10^5^ CAR-Tregs were co-cultured in 24-well non-tissue culture treated plates with a mixture of Treg and βlox5 media (1:1) in the presence of rIL-2 (100 IU/ml) for 48 hours. Cells in the plates were analyzed with inverted microscope, and images were taken.

### Terminal deoxynucleotidyl transferase–mediated dUTP nick end labeling assay

Coverslips coated with Poly-D-Lysine (Gibco) were seeded with βlox5 cells for 24 hours. Then 1x10^6^ CAR-Tregs were added and co-cultured with βlox5 cells for another 24 hours in mixed Treg and βlox5 media (1:1) in the presence of rIL-2 (100 IU/ml). For detection of apoptosis, the coverslips were stained using the *In situ* Cell Death detection kit (Roche). Details of TUNEL assay were provided in **Supplementary Materials and Methods**. The TUNEL-stained sections were analyzed using Leica SP8 White Light Confocal microscope with a 40X HC PL APO, CS2 oil objective lens (Stanford University Cell Sciences Imaging Facility). Images were evaluated for TUNEL staining using ImageJ. The percentage of TUNEL^+^ cells was determined as the percentage of TUNEL^+^ cells to total nuclei.

### Cytokine production assessment

4×10^5^ of bulk, CD39^+^ and CD39^−^ Treg cells were plated in 48 well plates and treated with Cell Activation Cocktail with Brefeldin A (Biolegend), a pre-mixed cocktail with optimized concentration of PMA (phorbol 12-myristate-13-acetate), ionomycin, and protein transport inhibitor (Brefeldin A) for 4–6 hours. Tregs were collected to stain and measure the intracellular production of IL-10, TNF-α, and IFN-γ by flow cytometry.

### Human pancreatic islet viability assessment using confocal microscopy

150 IEQ of human islets were co-cultured with 2x10^5^ CAR-Tregs (in triplicate) in a 96-well ultra-low attachment U-bottom plate for 48 hours. Subsequently, co-cultures were filtered with Round-Bottom Polystyrene Test Tubes with Cell Strainer Snap Cap (Falcon™, 5mL) and washed with PBS to remove Tregs and recover islets. Islets collected in a tube were then stained with LIVE/DEAD^®^ Viability/Cytotoxicity Kit for mammalian cells (Invitrogen) according to the manufacturer’s instructions. Images were obtained using a Leica SP2 confocal microscope.

### Human pancreatic islet gene expression assessment with real-time qPCR

150 IEQ of human islets were co-cultured with 2x10^5^ CAR-Tregs in a 96-well ultra-low attachment U-bottom plate for 24 hours. Subsequently, islets were recovered as described above. RNA was isolated from post-coculture human islets using the Allprep DNA/RNA micro kit (Qiagen). cDNA was synthesized using the Maxima First Strand cDNA synthesis kit (ThermoFisher) and gene expression was assessed by RT-qPCR using the Taqman Gene Expression Mix (ThermoFisher). The Taqman probes (Life Technologies) used were ACTIN*-*B, Hs4352667_m1; INSULIN, Hs00355773_m1.

### Human pancreatic islet cell viability assessment with flow cytometry

Islets were dispersed to single cell suspension with Accumax-Cell Aggregate Dissociation Medium (Invitrogen™) and co-cultured with CAR Tregs in 96-well ultra-low attachment U-bottom plate at a ratio of 1x10^5^ dispersed islet cells to 1x10^5^ Tregs for 48 hours. Cells were then collected and stained with CD3, CD4, HPi2, and HPx1 followed by 7-AAD/Annexin V Apoptosis Detection Kit (BioLegend) according to the manufacturer’s instructions. Samples were analyzed by flow cytometry.

### Mixed lymphocyte reaction suppression assay

Bead-enriched CD4^+^ T effector cells (Teff) from PBMCs were labeled with CellTrace™ Oregon Green™ 488 (CTOG, ThermoFisher). Autologous unmodified or CAR Tregs were labeled with CellTrace™ Violet Cell Proliferation Kit (ThermoFisher). PBMCs from HLA-mismatched and HLA-A2^+^ human donors were used as activator and antigen presenting cells. A Teff: PBMC ratio of 1:3 is used. Co-cultures were plated in 96-well U-bottom tissue culture plates (Falcon™). Teff proliferation was measured via CTOG 488-dilution of CD3^+^CD4^+^ lymphocytes on days 5 of co-culture by flow cytometric analysis.

### Genotype analysis

DNA was isolated from PBMC or Tregs for samples with QIAamp DNA Mini Kit (Qiagen) and array genotyped using the Illumina Global Diversity Array (GDA). Genotype quality control was performed using PLINK2 excluding variants with a low call rate (>2% missing genotypes) or not in Hardy-Weinberg equilibrium (HWE, p<1x10–6). After passing quality control, genotype dosage for the directly genotyped SNP rs10748643 were compared to sample CD39 expression and CAR Treg cytotoxicity in R.

### CD39 CRISPR/Cas9 knockout

A CD39-specific CRISPR/Cas9 knockout system (Gene Knockout Kit v2 - human - *ENTPD1*) was purchased from Synthego Corporation (Redwood City, CA, USA). In brief, FACS-sorted Tregs were grown in culture for 6–7 days with rhIL2 and anti‐CD3/CD28‐stimulation (Dynabeads™, ThermoFisher). Expanded Treg cells were harvested, and activation beads were removed prior to transfection. RNPs were preassembled *in vitro* by complexing premixed gRNAs to Cas9 following manufacturer’s instructions. The cells were mixed with the RNPs and electroporated with Neon™ Transfection System (ThermoFisher Scientific). 24 hours post-transfection, cells were stimulated and expanded again with Dynabeads™ at a cell-to-bead ratio of 2:1 for 2–3 days before CAR transfection. Knockout efficiency was assessed by flow cytometric analysis of CD39 proteins 4–5 days post-ransfection.

### Lentiviral overexpression of CD39 in bulk Tregs from CD39^low^ expressor

*ENTPD1* (CD39) was subcloned into pCDH EF1a MCS along with GFP separated by T2A. Lentivirus was packaged by transfection of a lentiviral vector, along with psPAX2 (plasmid 12260; Addgene) and pMD2.G (plasmid 12259; Addgene) expression vectors into HEK-293T cells using Lipofectamine2000 (ThermoFisher). Lentiviral particles were collected 48 and 72 hours after transfection and concentrated 300-fold with ultracentrifugation at 20,000 × g for 2 hours at 4°C. For lentiviral transduction, FACS-sorted Treg cells were activated with anti-CD3/anti-CD28 beads at a cell-to-bead ratio of 1:3 for two days. Cells were counted, plated, and lentiviral particles were added to the cells followed by spinoculation at 600 xg for 30 minutes at room temperature. Following this, complete media was added, and the cells were incubated at 37°C. After 7 days of culture expansion, CD39^+^GFP^+^ cells were sorted and re-stimulated with Dynabeads™ at a cell-to-bead ratio of 2:1 for 2–3 days before CAR transfection.

### Statistical analysis

Standard two-tailed Student’s *t* tests and nonparametric tests were performed to analyze statistical significance of the two groups. One-way and Two-way analysis of variance (ANOVA) tests were performed to examine the statistical significance of datasets with grouped analyses. Statistical significance was set at *P* < 0.05 in this study. All graphs and statistical analyses were completed using GraphPad Prism (ver.9.0.2).

## Data availability statement

The original contributions presented in the study are included in the article/**Supplementary Materials**. Further inquiries can be directed to the corresponding author.

## Ethics statement

The studies involving humans were approved by Stanford University Institutional Review Board (IRB) protocol #35453. The studies were conducted in accordance with the local legislation and institutional requirements. Written informed consent for participation in this study was provided by the participants’ legal guardians/next of kin.

## Author contributions

XW: Writing – review & editing, Writing – original draft, Visualization, Validation, Methodology, Investigation, Formal analysis, Data curation, Conceptualization. P-IC: Writing – review & editing, Writing – original draft, Visualization, Validation, Methodology, Investigation, Formal analysis, Data curation, Conceptualization. RW: Investigation, Writing – review & editing, Writing – original draft, Visualization, Methodology. MM: Formal analysis, Data curation, Visualization, Writing – review & editing, Writing – original draft, Methodology. VC: Writing – review & editing, Methodology. HY: Writing – review & editing, Methodology. YK: Writing – review & editing, Methodology. WH: Writing – review & editing, Methodology. SP: Validation, Writing – review & editing, Methodology. BI: Methodology, Writing – review & editing. AH: Writing – review & editing, Methodology. DS: Writing – review & editing, Resources, Methodology. ES: Writing – review & editing, Resources, Methodology. JS: Writing – review & editing, Resources, Methodology. DM: Resources, Writing – review & editing, Methodology. MB: Writing – review & editing, Resources, Methodology. SS: Formal analysis, Data curation, Writing – review & editing, Methodology. AG: Writing – review & editing, Supervision, Methodology. AP: Resources, Conceptualization, Writing – review & editing. SK: Writing – review & editing, Supervision, Funding acquisition, Conceptualization. KJ: Validation, Resources, Conceptualization, Writing – review & editing, Supervision, Methodology, Investigation. EM: Conceptualization, Writing – review & editing, Writing – original draft, Supervision, Project administration, Investigation, Funding acquisition.
