# Supplementary material for: CD39 delineates chimeric antigen receptor regulatory T cell subsets with distinct cytotoxic & regulatory functions against human islets
Source: Front Immunol. 2024 Jun 28;15:1415102. doi: 10.3389/fimmu.2024.1415102 (PMC11239501; doi:10.3389/fimmu.2024.1415102)
Supplement: Supplementary file 1 [file DataSheet_1.pdf]

Supplemental Materials and Methods

Cell culture conditions

Sorted Treg populations were cultured in X-VIVO™ 15 Chemically Defined Medium, with gentamicin (Lonza) supplemented with 10% heat inactivated human male AB serum (Sigma-Aldrich) and 10% L-glutamine (HyClone™), 10% sodium pyruvate (Gibco) and 10% non-essential amino acids (NEAA) (ThermoFisher) in the presence of recombinant human IL-2 (rIL-2, Hoffmann-LA Roche Inc) (100 IU/ml for $CD4^+$ T cells; 500 IU/ml for Tregs), and expanded using Dynabeads® Human T-Activator CD3/CD28 (ThermoFisher) according to the manufacturer's instructions. Tregs were cultured for 12 consecutive days, checked every 6 days for purity by flow cytometry. Treg purity was assessed by staining with anti-FoxP3 (Clone 206D, BioLegend) in eBioscience FoxP3 Transcription Factor staining buffer set (ThermoFisher). Flow cytometry data were collected on an LSRII (Becton Dickinson). FlowJo version v10 software (Treestar) was used for analysis.

βlox5 cells were expanded in low glucose (5.5mM) DMEM (Cellgro), supplemented with 10% FBS (HyClone™) and 1% penicillin-streptomycin (Gibco), 0.02% BSA (Sigma) and 1% non-essential amino acids (NEAA)(Fisher) and 15mM HEPES (Cellgro).

Terminal deoxynucleotidyl transferase–mediated dUTP nick end labeling (TUNEL) assay

Coverslips (12 mm High performance coverslip, Neuvitro) were coated with Poly-D-Lysine (Gibco) according to manufacturer's protocol. The coated sterile coverslips were placed into 12-well tissue culture plate using sterile forceps. $0.5x10^5$ βLox5 cells were seeded onto the cover slips for 24 hours. Then $1x10^6$ CAR-Tregs were added and cocultured with βlox5 cells for 24 hours in mixed Treg and βlox5 media (1:1) in the presence of rIL-2 (100 IU/ml). For detection of apoptosis, the coverslips were stained using the *In situ* Cell Death detection kit (Roche). Briefly, the coverslips were washed once gently with PBS, and then incubated with PE-conjugated HLA-A2 antibody at room temperature for 30 min. The coverslips were washed gently in PBS three times for 5 minutes each and then fixed in 4% paraformaldehyde (PFA) for one hours at room temperature. Samples were then permeabilized with 0.1% Triton X-100, 0.1% sodium citrate, for 2 minutes at room temperature. The slides were rinsed twice with PBS. 50 µl of TUNEL reaction solution (In Situ Cell Death Detection Kit, Roche) was added onto each coverslip and incubated for 1 hour at 37°C in the dark. Samples were embedded with DAPI Fluoromount-G (SouthernBiotech) prior to analysis. Negative controls for TUNEL staining included only 50ul label solution. Finally, the sections were analyzed using Leica SP8 White Light Confocal microscope with a 40X HC PL APO, CS2 oil objective lens (Stanford University Cell Sciences Imaging Facility). Images were evaluated for TUNEL staining using ImageJ. The percentage of TUNEL positive cells was determined as the percent of TUNEL positive cells to total nuclei**.**

Table S1 Antibodies used for flow cytometry.

Target	Clone	Fluorochrome	Vendor	RRID
CD3	OKT3	Alexa Fluor 488	BioLegend	AB_571877
CD3	OKT3	APC	BioLegend	AB_1937212
CD3	OKT3	BV785	BioLegend	AB_2563507
CD4	SK3	Alexa Fluor 700	BioLegend	AB_2563150
CD4	OKT4	Alexa Fluor 488	BioLegend	AB_571939
CD8	SK1	BUV805	BD Biosciences	AB_2833078
CD8	SK1	APC/Cyanine7	BioLegend	AB_2044006
CD25	BC96	PE/Dazzle™ 594	BioLegend	AB_2734260
CD25	2A3	BUV395	BD Biosciences	AB_2738556
CD127	A019D5	PerCP/Cyanine5.5	BioLegend	AB_10897104
CD39	eBioA1	BV605	BioLegend	AB_2750430
CD45RO	UCHL1	BUV395	BD Biosciences	AB_2744410
CD45RO	UCHL1	Alexa Fluor 488	BioLegend	AB_528823
CD62L	DREG-56	PE-Cy7	BioLegend	AB_830801
PD-1	NAT105	BV785	BioLegend	AB_2721562
Tim-3	F38-2E2	PE/Dazzle™ 594	BioLegend	AB_2565886
LAG-3	11C3C65	BV650	BioLegend	AB_2632951
FOXP3	206D	BV421	BioLegend	AB_2565972
IL-10	JES3-19F1	APC-R700	BD Biosciences	AB_2870004
IFN-γ	4S.B3	BV605	BioLegend	AB_2563881
TNF-α	Mab11	APC	BioLegend	AB_315264
IL-4	MP4-25D2	PE	BioLegend	AB_315129
Granzyme B	GB11	Alexa Fluor 647	BioLegend	AB_2566333
Granzyme B	QA16A02	APC/Fire™ 750	BioLegend	AB_2728377
HLA-A2	BB7.2	PE	BioLegend	AB_1877227

HLA-A2	BB7.2	APC	BioLegend	AB_2561567
PD-L1	29E.2A3	PE-Cy7	BioLegend	AB_2561687
CD209	9E9A8	Alexa Fluor 647	BioLegend	AB_1186092
CD14	M5E2	APC/Fire™ 750	BioLegend	AB_2632660
CD163	GHI/61	PerCP/Cyanine5.5	BioLegend	AB_2228986
CD16	3G8	BUV395	BD Biosciences	AB_2744293
CD206	19.2	BUV805	BD Biosciences	AB_2871334
CD11c	Bu15	BV421	BioLegend	AB_2564485
CD11b	ICRF44	BV605	BioLegend	AB_2562021
CD86	IT2.2	BV785	BioLegend	AB_2616794
HLA-DR	L243	PE/Dazzle™ 594	BioLegend	AB_2563646
HPx1	HIC0-3B3	DyLight 405	Novus Biologicals	
HPi2	HIC1-2B4.2B	Alexa Fluor 647	Novus Biologicals	

Supplemental Figures

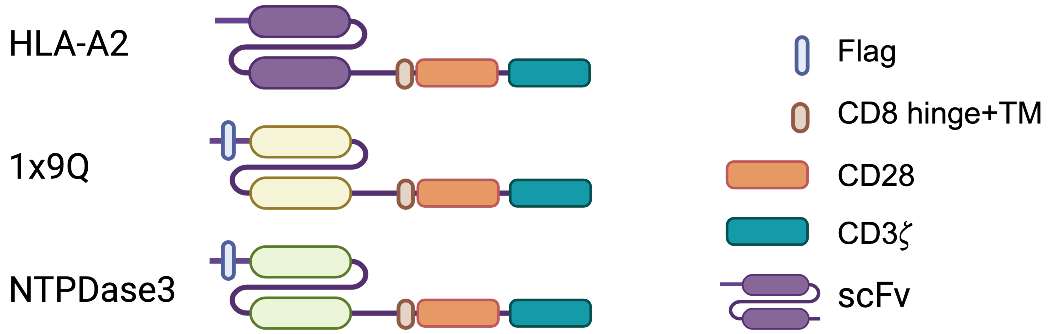

Figure S1 | Domain structures of HLA-A2, 1x9Q and NTPDase3 CARs.

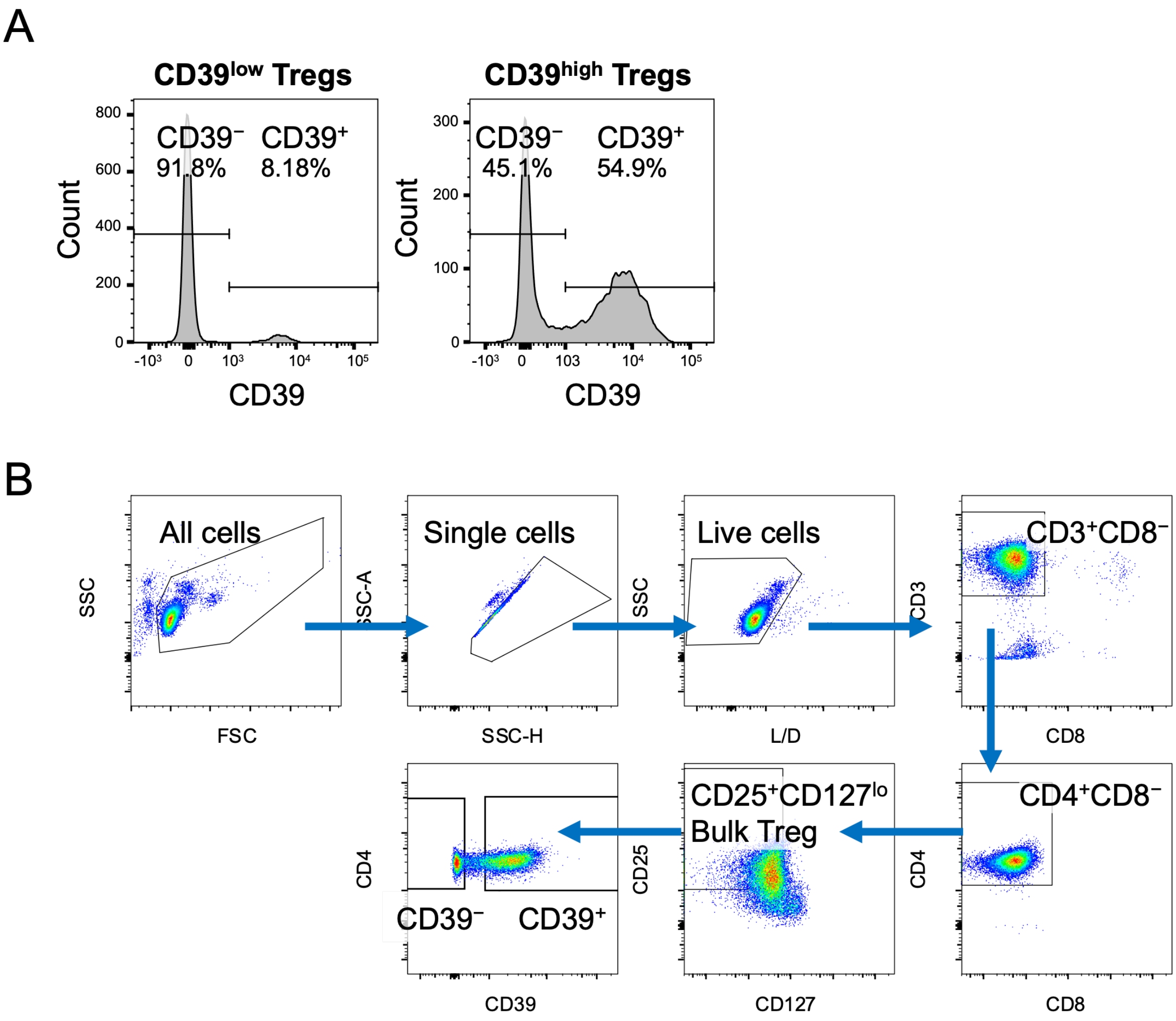

Figure S2 | Treg subset definition and FACS sort gating hierarchy

(A) Bulk Tregs with low frequency of $CD39^{+}$ Treg cells is defined as $CD39^{low}$ Tregs, the others with high frequency of $CD39^{+}$ Treg cells is defined as $CD39^{high}$ Tregs. **(B)** Bulk Tregs were gated as $CD3^{+}CD4^{+}CD8^{-}CD25^{+}CD127^{low}$, and then sorted into $CD39^{+}$ and $CD39^{-}$ Treg subsets.

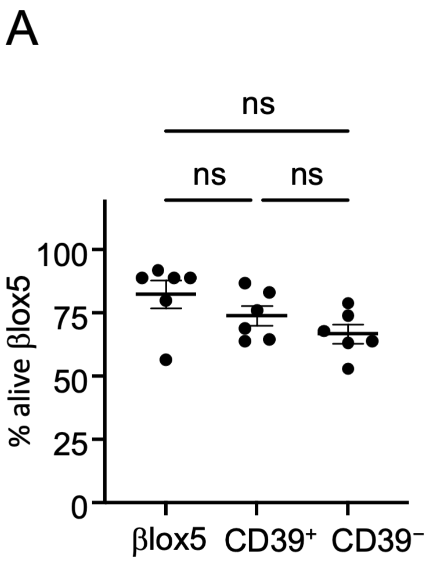

Figure S3 | Introduction of HLA-A2-CAR induced cytotoxicity of Tregs to βlox5 cells. (A) Expanded but unmodified CD39+ and CD39− Tregs were co-cultured with βlox5 cells for 24 hours. Cells were harvested, and viability was assessed by flow cytometric analysis of 7-AAD and Annexin V staining. Percent of viable cells (AnnexinV^{-} 7-AAD^{-}) was graphed (mean ± SEM, n=6). **(B)** Expanded bulk Tregs were transfected with 1x9Q or A2-CARs and co-cultured as above. Viability was assessed and analyzed as in A. (mean ± SEM, n=6). * $P < 0.05$, ** $P < 0.01$, *** $P < 0.001$, **** $P < 0.0001$; ns, not significant by one-way ANOVA, Tukey's multiple comparisons test.

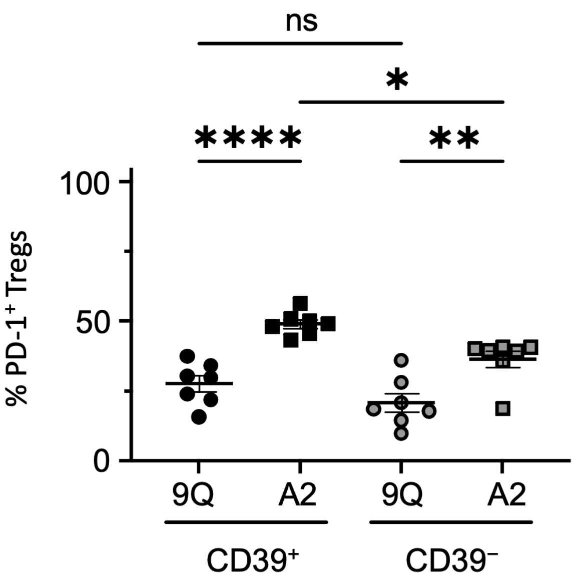

Figure S4 | HLA-A2 CAR expressing Tregs increased PD-1 expression when co-cultured with βlox5 cells. Expanded CD39$^+$ and CD39$^-$ Tregs were transfected with 1x9Q or A2-CARs and co-cultured with βlox5 cells for 24 hours. PD-1 expression was measured in live Treg cells by flow cytometry. Percent of PD-1$^+$ Treg cells was graphed (mean ± SEM, n=7). * $P < 0.05$, ** $P < 0.01$, *** $P < 0.001$, **** $P < 0.0001$; ns, not significant by one-way ANOVA, Tukey's multiple comparisons test.

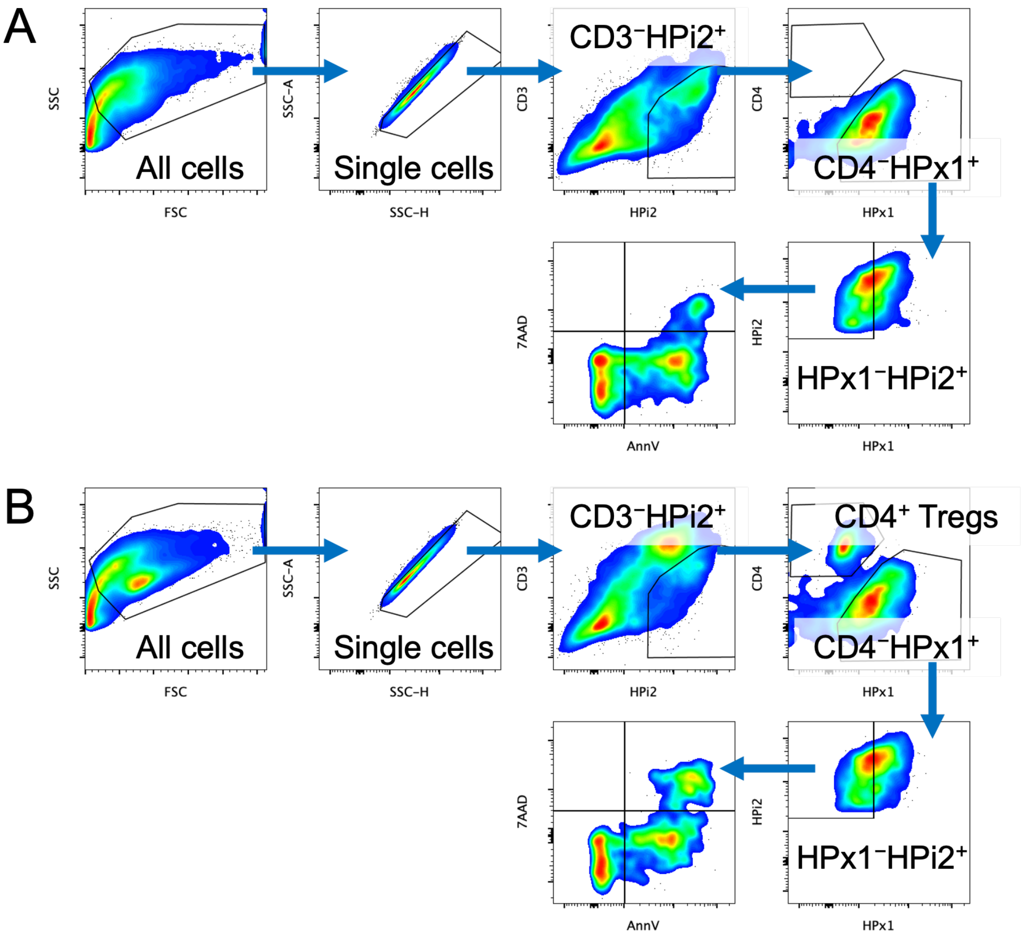

Figure S5 | Gating hierarchy for apoptosis analysis of islet endocrine cells.

(A) Dispersed islet cells only were stained for CD3, CD4, HPx1 and HPi2, and then stained with 7AAD/Annexin V to assess apoptosis. Islet endocrine cells were identified as $CD3^{-}$ $CD4^{-}$ $HPx1^{-}$ $HPi2^{+}$ cells. **(B)** Dispersed islet cells were co-cultured with CAR Tregs for 48 hours and then analyzed as in **A.**
